# Cognitive Behavioral Group Therapy Reduces Stress and Improves the Quality of Life in Patients with Parkinson’s Disease

**DOI:** 10.3389/fpsyg.2016.01975

**Published:** 2017-01-04

**Authors:** Anousha Hadinia, Antonia Meyer, Viviane Bruegger, Florian Hatz, Karolina Nowak, Ethan Taub, Elisabeth Nyberg, Rolf-Dieter Stieglitz, Peter Fuhr, Ute Gschwandtner

**Affiliations:** ^1^Department of Neurology, University Hospital of BaselBasel, Switzerland; ^2^Department of Neurosurgery, University Hospital of BaselBasel, Switzerland; ^3^Department of Psychiatry, University Hospital of BaselBasel, Switzerland; ^4^Department of Psychology, University of BaselBasel, Switzerland

**Keywords:** cognitive behavioral therapy, Parkinson’s disease, stress, quality of life, non-motor symptoms, cognitive behavioral group therapy

## Abstract

**Objective:** The aim of this study is to compare a cognitive behavioral group therapy (CBT) with a health enhancement program (HEP) for stress reduction and the impact on quality of life (QoL) in patients with Parkinson’s disease (PD).

**Method**: Thirty patients with PD participated in the study: 16 received CBT including stress-reducing elements and 14 took part in a HEP. The two groups did not differ significantly in their baseline demographic characteristics. The patients in both groups underwent weekly sessions of 2 h duration for 9 weeks. The *Parkinson’s Disease Questionnaire* with 39 items (PDQ-39), the *Burden Questionnaire for Parkinson’s Disease* (translated from the original German: *Belastungsfragebogen für Parkinsonpatienten* (BELA) and the *Disease-Related Questionnaire* [*Fragebogen zur krankheitsbezogenen Kommunikation* (FKK)] were used for assessment. Ratings were completed at baseline and after 9 weeks (immediately after the last treatment session).

**Results**: The patients in the CBT group achieved significantly better BELA, FKK and PDQ-39 scores (*p* < 0.05). Subscale analysis revealed that the scores on the BELA subscales “emotional well-being” and “somatic motor function” contributed significantly to stress reduction (*p* < 0.05). The FKK revealed significant improvement in social skills in the CBT group (*p* < 0.05).

**Conclusion**: Cognitive Behavioral Group Therapy appears to be an effective way for patients with PD to lessen stress and improve their quality of life.

## Introduction

Parkinson’s disease (PD) is a neurodegenerative disease characterized by both motor and non-motor dysfunction. The motor manifestations including rigor, tremor, postural instability, and freezing of gait are well described and commonly recognized (Shulman et al., 2002; Chaudhuri et al., 2006). The non-motor manifestations such as fatigue, apathy, depression, anxiety, and sleeplessness have recently attracted increased scientific interest and appear to have a greater impact on the quality of life (QoL) than the motor manifestations (Richard, 2005; Martinez-Martin, 2011).

Martínez-Martín (1998, pp. 2–6) defined the health-related QoL of patients with PD as “the perception and evaluation, by patients themselves, of the impact caused on their lives by the disease and its consequences.”

Depression (Weintraub et al., 2004; Martinez-Martin, 2011; Jones et al., 2015) and anxiety have been found to be associated with a low QoL (Jones et al., 2015). Soh et al. (2011) analyzed 29 articles that examined predictors of QoL in patients with PD. Besides motor symptoms, disease severity, and demographic variables, non-motor symptoms such as apathy, depression, and anxiety were found to predict QoL. Depression and anxiety are also negatively associated with the motor symptoms of PD (Dissanayaka et al., 2011) and the progression of the disease (Schrag et al., 2000; Leentjens et al., 2013).

Cognitive behavioral therapy (CBT) is an effective treatment for depression, anxiety, and phobias, in both individual and group therapy settings (Hofmann et al., 2012). Even in elderly patients (Peng et al., 2001; Ceri, 2007) and in patients with neurological diseases, positive effects such as a reduction of depressive symptoms have been reported (Mohr et al., 2001, 2004). Until now, studies have been focusing on the treatment of the non-motor symptoms such as depression and anxiety rather than the improvement of QoL. Dobkin et al. (2011) examined the impact of individual CBT on depression and anxiety on 80 patients with PD during 10 weeks. The control group only received four unspecific, short phone calls. Patients receiving CBT have shown significantly less symptoms of depression and anxiety than patients in the control group. This study has shown that individual CBT lessens depressive symptoms and anxiety in patients with PD. Patients have also reported an improvement of their QoL (Dobkin et al., 2011, 2012). A statistical analysis of QoL has not been assessed. Based on this study, Dobkin et al. (2012) discerned predictive factors of the effect of CBT on depression and anxiety. The inclusion of caregivers predicts the effect of the treatment (Dobkin et al., 2012). Considering the negative impact of non-motor symptoms on QoL, a focus on the improvement of QoL could be advantageous.

There have been only a few studies of the use of CBT to treat QoL and stress in patients with PD. A high stress level reinforces and establishes motor symptoms (Metz, 2007; Hemmerle et al., 2012). CBT aims to identify the stress level and modify negative cognitive beliefs and behaviors (Beck, 1979). Therefore it might yield greater improvement than drug treatment alone, and any type of effective treatment would be of considerable clinical significance in view of the high prevalence of PD (1–2%) (Moreno et al., 2013). Group CBT is more cost-effective and has similar effects as individual CBT (McDermut et al., 2001). In addition there is a lack of studies that use an active control group, which receives the same frequency of sessions. The health enhancement program (HEP) of MacCoon et al. (2012) has been developed for the purpose of building an active control intervention for a mindfulness based stress study. To fill these gaps in research, the present study examines a randomized, controlled trial of CBT for reducing stress and improving QoL in patients with PD, with HEP as a control intervention.

## Materials and Methods

### Subjects

**FIGURE 1:**
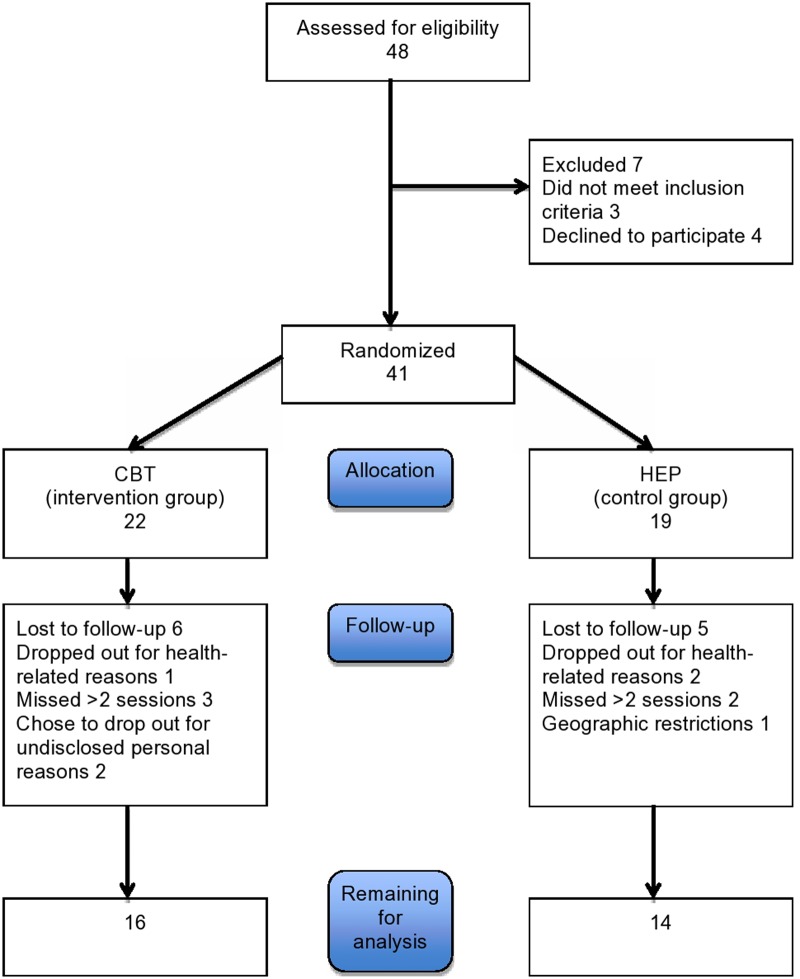
**Participant flow**.

Patients were recruited through advertisement on a flyer at the University of Basel and from the movement disorder clinic. Patients who met the *UK Parkinson’s Disease Brain Bank Criteria* for PD were considered eligible for the study. Patients with severe dementia or physical impairment or with severe neurologic or psychiatric deficits were excluded. Forty one Patients were recruited. Eleven patients dropped out either due to health related problems or because they missed more than 2 sessions or refused to continue. Of the remaining 30 patients, 16 were assigned to the CBT group and 14 to the control (HEP) group (see **Figure 1**). Their baseline characteristics are given in **Table 1** The two groups were comparable in age, educational level, and sex. All patients gave written informed consent to participation. The study was approved by the local ethics committee, the Ethikkommission Zentral- und Nordwestschweiz (verification number EKNZ: 294/13).

**Table 1:** Baseline characteristics of the two study groups (means and standard deviations).

	Intervention group CBT	Control group HEP	*p^a^*
*Number of patients*	16	14	
Age (years)	65 (8.7)	67 (11)	0.62
Sex (number female)	4	3	0.82
Education (years)	15 (3.4)	15 (3.5)	0.89
Medication (LED)	630 (460.1)	468 (299.7)	0.32
DBS (number who underwent DBS surgery)	6	3	0.34
Duration of disease (years)	15 (8.9)	18 (8.6)	0.96
UPDRS III	88 (71.1)	94 (89)	0.33
MMSE	29 (1.6)	29 (0.8)	0.41
MOCA	26 (2.4)	26 (2.3)	0.83
BDI	8 (6)	8 (4.7)	0.48
BAI	12 (8.1)	10 (8.3)	0.84
AES	32 (9.7)	30 (6.5)	0.59
PFS	49 (12.1)	39 (17.8)	0.16
ESS	8 (5.9)	6 (4.6)	0.38


*CBT, Cognitive Behavioral Therapy; HEP, Health Enhancement Program; DBS, Deep Brain Stimulation; UPDRS III, Unified Parkinson’s Disease Rating Scale cognitive deficits; MMSE, Mini Mental State Examination; MOCA, Montreal Cognitive Assessment; BDI, Beck Depression Inventory; BAI, Beck Anxiety Inventory; AES, Apathy Evaluation Scale; PFS, Parkinson Fatigue Scale; ESS, Epworth Sleepiness Scale. Data are all means unless otherwise indicated. The statistical tests used were analysis of variance (ANOVA) and the χ^*2*^ test (for sex and DBS); ^a^Significant at the 5% level.*

### Study Design

Upon inclusion in the study, the patients were allocated to the two groups by a computer-generated randomization designed to yield groups that were balanced in terms of size, age, educational level, and sex. The patients were not told to which group they had been allocated or what treatment they would receive. The data were analyzed in blinded fashion.

### Treatments

All patients in both groups underwent weekly treatment sessions for the duration of 2 h for 9 weeks. These sessions took place at the University Hospital of Basel, Switzerland. For CBT, a modification of the manual of Ellgring et al. (2006) was used. Each CBT session included different content: (1) self-observation, (2) stress reduction, (3) relaxation and enjoyment, (4) expression of the illness, (5) sadness and depression, (6) seeking help, (7) role of relatives, (8) relationship with relatives, (9) summary of all sessions. Family members participated in two of the sessions. After every session, the new skills had to be practiced once as homework. CBT was carried out by a clinical psychologist and a trainee in clinical psychology.

**FIGURE 2:**
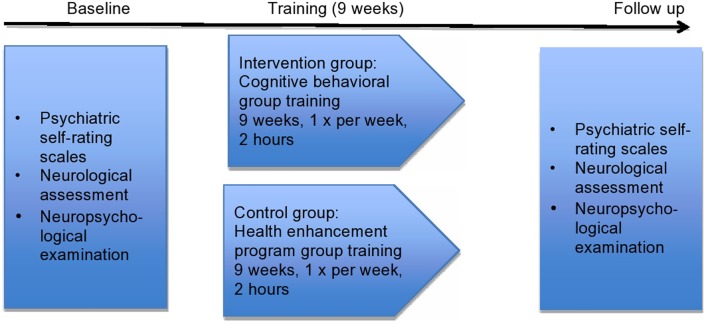
**Study design**.

The control group received the HEP (MacCoon et al., 2012) with the following contents in each session: (1) and (2) music therapy, (3, 4, and 5) physical activity, (6 and 7) dietary counseling, (8) medical information by a physician and (9) summary of all sessions. Clinical psychologists and trainees in clinical psychology conducted all nine sessions in the control group. The study design is illustrated in **Figure 2**.

### Measurements

The patients were asked to complete self-rating questionnaires in German at baseline and after treatment.

#### PDQ-39

The *Parkinson’s Disease Questionnaire 39* (PDQ-39; Jenkinson et al., 1997) was used to assess QoL. The 39 items scale (e.g., “I have had difficulties to walk 1 km in the last month”) are answered on a 5 point Likert scale (never; seldom; sometimes; often and always). The possible total score ranges from 0 to 156. Lower values are indicating higher QoL.

#### Belastungsfragebogen für Parkinsonpatienten

Stress was assessed with *the Burden Questionnaire for Patients with Parkinson’s disease*, (original name: *Belastungsfragebogen für Parkinsonpatienten* (BELA); Macht and Ellgring, 2003). The questionnaire consists of 34 Items, which are answered on a 4 point Likert scale before a training (reaching from 0-3, e.g., “I feel anxious: not at all, a little, mostly, always”). After the training, patients assessed the change of the problem on a 5 point Likert scale ranging from -2 to 2 (became much worse, a little worse, stayed the same, became a little better and became much better). A negative score indicates an exacerbation of the burden after the training (min. score -40). A score of 0 indicates no change and a positive score shows a positive change after the training (maximum score 40).

#### Fragebogen zur Krankheitsbezogenen Kommunikation

The *Questionnaire for Disease-Related Communication*, [original name: *Fragebogen zur krankheitsbezogenen Kommunikation* (FKK); Macht and Ellgring, 2003] was also used to assess stress. Similar to the BELA, a 4 point Likert scale ranging from 0 to 3 was used to answer the 20 items before the training. The same 5 point Likert scale as described above was used to measure change after training. The minimal to maximal score before the training ranges from 0 to 60, and after the training from -40 to 40.

To detect possible confounding variables, the patients and their family members and neurologists were asked to supply data of the following types:

#### UPDRS III

Based on 18 Items, the *Unified Parkinson’s Disease Rating Scale* (UPDRS III; Fahn and Elton, 1987) assesses motor involvement in patients with PD. On a 5 point Likert scale (ranging from 0 to 4) patients evaluate their difficulties by motor impairment in daily activities (normal; slight; mild; moderate and severe). While 0 points indicates no difficulties, the maximum of 72 points implies severe difficulties.

#### Mini Mental State Examination

The *Mini Mental State Examination* (MMSE; Folstein et al., 1979) measures the occurrence and severity of cognitive impairment. In 11 tasks evaluated by a professional, patients can reach a maximum score of 30 points, meaning no impairment at all. Scores between 20 and 26 indicate a mild cognitive impairment, between 10 and 19 a moderate and below 9 a severe cognitive impairment.

#### Montreal Cognitive Assessment

In addition to the MMSE, the *Montreal Cognitive Assessment* (MOCA; Nasreddine et al., 2005) also assesses cognitive deficits. The minimum to maximum score of the 11 tasks, instructed by a professional, ranges from 0 to 30. A score below 23 indicates a cognitive impairment.

#### BDI-II

Depressive symptoms were assessed with the German version of *Beck Depression Inventory II* (BDID; Hautzinger et al., 2006). Patients were asked to fill in 21 items. The 4 point Likert scale ranges from 0 to 3 (“I do not feel sad”; “I am sad all the time”; “I am so sad and unhappy I can’t stand it”). Total scores range from 0 to 63. A higher score implies more depressive symptoms.

#### Beck Anxiety Inventory

The German version of the *Beck Anxiety Inventory* (BAI; Beck et al., 1988) measures in 21 items on a 4 point Likert scale the occurrence of sensations related to anxiety (e.g., not at all; a little; moderate and many). The minimum score is 0, indicating not feeling any anxiety at all, whereas the maximum score is 63 meaning a high sensation of anxiety.

#### Apathy Evaluation Scale

The *Apathy Evaluation Scale* (AES; Lueken et al., 2006) is a self-rating questionnaire, which evaluates in 18 items the appearance and severity of apathy. On a 4 point Likert scale patients evaluate if and how severe the given situation applies to them (not at all; a little; moderate and many). Item score ranges from 0 to 3. The total score ranges from 0 to 53, with a higher value indicating a higher frequency of apathy.

#### Parkinson Fatigue Scale

To measure the appearance of fatigue, patients filled in the *Parkinson Fatigue Scale* (PFS; Brown et al., 2005). The 5 point Likert scale of the 16 items ranges from 0 to 4 (does not apply at all; does not apply; whether nor; does apply a little and applies a lot). A total score of 0 indicates no appearance of apathy, while a higher value implies a higher frequency of apathy. The maximum score is 64.

#### Epworth Sleepiness Scale

To evaluate the quality of sleep, the *Epworth Sleepiness Scale* (ESS; Johns, 1991) was used. Eight situations, which can be evaluated on a 4 point Likert scale in a self-rating fashion, are described. The item scale ranges from 0 (no likelihood that situation emerges) to 3 (high likelihood that situation emerges). The total score has a minimum of 0 and a maximum of 24 points. A higher score indicates a more severe sleepiness.

We also performed a battery of neurological tests on all patients at baseline and on follow-up; the results of these tests will be the subject of a further publication.

### Confounding Variables

Statistical analyses were carried out with SPSS (version 22.0.0.0). Individual missing items were replaced by mean values; entire missing questionnaires were not included.

The two study groups were compared at baseline to detect potential confounding variables.

Analysis of variance (ANOVA) and chi-squared tests were used to detect intergroup differences in age, disease duration, educational level, medication (levodopa equivalent dose), sex, motor manifestations (UPDRS III), cognitive deficits (MMS, MOCA), depression (BDI), anxiety (BAI), apathy (AES), fatigue (PFS), and sleepiness (ESS).

### Intergroup Comparisons of Changes in Scores

The effect of training was assessed by the differences between the patients’ PDQ-39 scores before and after training. 0 to 3 points can be assigned for each item of the PDQ-39. In the BELA and the FKK, the patients themselves were asked to rate the effect of training on each item (got worse, a little worse, no change, a little better, much better). Item scores accordingly ranged from -2 to 2. The scales of the PDQ-39 were inverted for consistency in analysis, so that a high score would indicate an improvement in all scales.

MANOVA was performed to compare general effects of training in both groups on the basis of the overall sum difference in the PDQ-39 and the change score of the sum in BELA and FKK.

Because of the small sample size and the large number of subscales, the differences in the PDQ-39, BELA and FKK subscales were calculated with ANOVA.

### Predictor Variables

To find possible variables that might predict outcome variables in the intervention group, a Spearman correlation was generated between outcome variables and all possible variables at baseline. The putative predictors were subjected to multiple regression analysis.

## Results

### Baseline Characteristics of the Two Study Groups

The characteristics of the two study groups at baseline are shown in **Table 1**. We considered any findings with *p* < 0.05 to be statistically significant. The two groups did not differ significantly at baseline with respect to age, sex, educational level, disease duration, fatigue, apathy, sleepiness, depression, anxiety, or MMSE and UPDRS III scores.

### Intergroup Differences of Changes in Stress and the Quality of Life

These results are shown in **Table 2** (MANOVA). The two groups differed significantly with respect to stress as assessed by the BELA (*p* = 0.026), with a large effect size (η^2^ = 0.139) and likewise as assessed by the FKK (*p* = 0.037, η^2^ = 0.118) in favor of the intervention group. Therefore, the impact of CBT on patients with PD is more effective than the impact of HEP. The control group showed a negative change score in BELA, which indicates an increase in stress after receiving the HEP.

**Table 2:** Change scores relating to stress and quality of life.

	Change score			
	
	CBT mean (SD)	HEP mean (SD)	*p*	Effect size (η^2^)
BELA	5.56 (11.48)	-2.75 (9.36)	0.03^a^	0.14
FKK sum	4.68 (5.98)	1.07 (2.28)	0.04^a^	0.12
PDQ-39 sum	5.31 (13.94)	3.25 (6.30)	0.03^a^	0.13


*BELA, *Belastungsfragebogen für Parkinsonpatienten;* FKK, *Fragebogen zur Krankheitsbezo-genen Kommunikation;* PDQ-39, Parkinson’s Disease Questionnaire. The statistical test used was multiple analysis of variance (MANOVA). A positive mean implies a benefit; ^a^Significant at the 5% level.*

The sum score of the PDQ-39 differed significantly between the groups (*p* = 0.03), indicating an improved QoL after CBT training, with a large effect size (η^2^ = 0.13).

### Intergroup Differences of Changes in Stress and Quality of Life Subscales

Subscale analyses were performed to determine which subscales made the greatest contributions to the observed intergroup differences. As shown in **Table 3**, score changes on the “emotional well-being” and “somatic motor function” subscales of the BELA were significantly better in the CBT group than in the HEP group after training — for emotional well-being, *p* = 0.033, η^2^ = 0.124; for somatic motor function, *p* = 0.039, η^2^ = 0.115. These two effects were of medium strength. Also, in these two subscales, there is a negative effect for the HEP group, indicating a deterioration of perceived emotional wellbeing and somatic motoric functioning after the training for the control group. There was no significant difference between groups in score changes on the “cognitive ability” subscale. As for the FKK subscales, the CBT group did significantly better on the social skills subscale than the HEP group (*p* = 0.031), meaning that skills which enable social interactions, with a nearly large effect size (η^2^ = 0.127). There was no significant difference in the social burden subscale (see **Table 3**).

**Table 3:** Change score in subscales of stress and quality of life.

	Change score			
	
	CBT mean (SD)	HEP mean (SD)	*p*	η^2^
**BELA**				
Emotional	3.56 (6.14)	-0.38 (4.09)	0.033^a^	0.124
Somatic motor function	1.18 (3.70)	-1.38 (3.81)	0.039^a^	0.115
Cognitive	0.50 (1.50)	-0.30 (0.94)	0.054	0.096
**FKK**				
Social burden	1.81 (2.80)	0.38 (1.19)	0.06	0.09
Social skills	2.94 (3.71)	0.69 (1.25)	0.03^a^	0.13
**PDQ-39**				
Mobility	5.19 (17.82)	3.42 (9.47)	0.07	0.08
Daily activities	6.80 (17.04)	1.67 (9.37)	0.07	0.09
Emotional well-being	0.50 (7.80)	1.33 (7.84)	0.27	0.01
Stigma	0.31 (11.32)	0.50 (10.86)	0.43	0.00
Social support	0.13 (19.43)	2.75 (5.45)	0.33	0.01
Cognition	4.81 (12.73)	0.42 (12.23)	0.14	0.04
Communication	4.70 (18.42)	3.58 (11.03)	0.42	0.00
Physical discomfort	3.67 (13.93)	8.33 (10.03)	0.17	0.03


*BELA, *Belastungsfragebogen für Parkinsonpatienten;* FKK, *Fragebogen zur Krankheits-bezogenen Kommunikation;* PDQ-39, Parkinson‘s Disease Questionnaire.Tests were Analysis of Variance (ANOVA). A positive mean implies a benefit; ^a^Significant at the 5% level.*

There were no significant intergroup differences between score changes on any of the PDQ-39 subscales, such as “mobility,” “daily activities,” “emotional wellbeing,” “stigma,” “social support,” “cognition,” “communication” and “physical discomfort.” When divided in subscales, the PDQ-39 does not show a greater improvement in QoL for the CBT group compared to the HEP group.

### Predictors

A correlation matrix was conducted to find possible predictor variables in the intervention group. The change in the overall score of PDQ-39 is correlated significantly with disease duration (*r* = 0.527; *p* = 0.036) and age (*r* = 0.507; *p* = 0.045). The correlation between the initial UPDRSIII score and outcome in terms of the change in the overall PDQ-39 score did not reach statistical significance (*r* = 0.472; *p* = 0.065). Variables were analyzed in a multiple regression for PDQ-39 demonstrating that disease duration influences the training effect in terms of QoL, if separately analyzed (β = 0.53; *R^2^* = 0.23). The significance of disease duration disappears if integrated in one model with age. Youth predicts a better effect of training on QoL; i.e., younger patients have a greater improvement in QoL after group CBT (β = 0.56; *R^2^* = 0.49) (**Table 4**).

**Table 4:** Multiple regression on PDQ-39 measured in intervention group (*n* = 16).

Independent variable	*B* (*SE*)	β	*Corr. R^2^*	*F*
Model1			0.23	5.56
Disease Duration	0.11 (0.04)	0.53^a^		
Model2			0.49	8.12
Disease duration	0.07 (0.04)	0.33		
Age	0.31 (0.31)	0.56^a^		
Model3			0.52	6.35
Disease duration	0.05 (0.04)	0.24		
Age	0.76 (0.32)	0.47^a^		
UPDRS III	0.45 (0.33)	0.28		


*PDQ-39, *Parkinson’s Disease Questionnaire 39*; UPDRS III, *Unified Parkinson’s Disease* Rating Scale cognitive deficits; ^a^Significant at the 5% level.*

## Discussion

In this study, we examined whether cognitive behavioral group therapy lessens stress and improves QoL in PD patients more effectively than a HEP. We found that it does, and that the effect is mostly accounted for by a worsening of BELA and PDQ-39 scores in the control (HEP) group. Similar findings were seen on all subscales except for the communication and physical discomfort subscales of the PDQ-39, on which all patients showed a decline. These results are comparable to earlier findings: Tiihonen et al. (2008) analyzed the effect of group CBT versus no treatment in PD patients with PD and found that the CBT patients die significantly better in terms of QOL as assessed by the PDQ-39, the difference being accounted for by a worsened QoL in the control group.

When interpreting the findings of this and other trials involving PD patients, one must bear in mind the progressive nature of the disease. The control group received physiotherapy, music therapy, and dietary counseling. The CBT training was intended to improve the patients’ emotional and social skills, so that they would suffer less from stress due to the manifestations of PD and would achieve a higher QoL despite progression of the disease. Patients in the CBT group received training that was focused on coping with the disease the development of self-efficacy (belief in one’s own ability to perform tasks and reach goals). Previous studies have revealed that self-efficacy contributes to a high QoL and a low stress level (Tiihonen et al., 2008). We think the worsening QoL in the control (HEP) group of this study may well have been due in large part to these patients’ increased distress over their progressive physical limitations. The BELA consists of questions about the emotional burden resulting from a variety of problems (anxiety, etc.), rather than these problems themselves. As HEP is focused on physical training rather than emotional burden reduction, one may speculate that the patients in our control group may even have experienced the HEP training itself as stressful, which, in turn, may have contributed to or accounted for their lower BELA change score. Likewise, in the PDQ-39, many of the items whose frequency the patients are asked to estimate are themselves feelings, such as worry about other people’s reactions. In this case as well, HEP training is not specifically directed against such items and could conceivably even make them worse.

Both groups reported increased physical discomfort after training. This shows that the CBT group did not experience better physical well-being through the development of strategies to deal with the disease itself, and that the HEP intervention did not improve the physical manifestations of the disease sufficiently to improve physical comfort. Perhaps coping with the disease is more important than direct treatment of its manifestations. In one study, a higher optimism score was correlated with lesser severity of disease (Shifren, 1996), indicating that disease severity is influenced by the patient’s perception. It follows that a treatment focused on a change in attitude toward the disease could be beneficial.

This study also shows that younger patients with PD show a larger improvement in QoL after CBT than older patients. The older patients in this study had generally suffered from PD for a longer time (*p* = 0.026); the effect of disease duration as a predictor disappears in the regression model with age. Younger patients, who generally have had the disease for a shorter time and have more years left to live, may well be better motivated to cope with the disease than older ones. Stanley et al. (2009) reported in their study using CBT for treating general anxiety disorder (GAD), greater effect sizes in younger patients than in older ones. To minimize this effect, Laidlaw et al. (2008), developed CBT concept specifically for elderly patients. The authors suggested to focus on issues that are explicitly related to this age, such as physical health, role characteristics, losses and relationship to younger generations. Even though we did have age related issues in the CBT treatment of this study, they were not discussed deeper. Instead we focused on disease-related issues where patients learned to restructure the negative associations with the disease, (e.g., being ashamed of it).

CBT activates the prefrontal cortex that is responsible for the process and regulation of emotions (Goldapple et al., 2004). This effect can be applied to any patient; therefore it can be used for a variety of patient groups. The review article of (Berardelli et al., 2015) shows that previous studies examining movement disorders found a reduction of depressive symptoms and an increase of QoL after receiving CBT. In a study using CBT on patient’s with multiple sclerosis, patients have shown a better QoL after the treatment. This effect was mediated by depression (Cosio et al., 2011). These findings show, that CBT can generally have an impact on QoL in patient’s with physical diseases.

The small scale of this study limits the interpretation of its findings. Because of the small number of observations, MANOVA could not be applied to all variables, and ANOVA had to be used instead, yielding less detailed findings. If more patients had been included in this study, its findings would have been more robust.

Moreover, this study did not include either a group of healthy subjects without PD or a group of PD patients who did not receive any training at all. Either or both of these negative controls might have revealed general training effects that this study, as performed, could not address. For future studies, a larger number of observations as well as a group of healthy participants would be necessary to reveal well funded statements.

With these limitations, this study indicate the possibility that CBT lessened stress and improved QoL to a greater extent than HEP in – particularly younger – patients with PD. We conclude that group CBT might be a promising treatment for preventing increased stress and a declining QoL in patients with PD.

## Author Contributions

AH: analysis and interpretation of data, acquisition of data. AM: study design, acquisition of data, and interpretation of data. VB: acquisition of data, interpretation of data. FH: acquisition of data, critical revision of manuscript. KN: study design, acquisition of data, and interpretation of data. ET: contribution of core ideas, critical revision of manuscript for important intellectual content. EN: supervision, critical revision of manuscript. R-DS: study concept, critical revision of manuscript. PF: study concept, acquisition of data, and critical revision of manuscript. UG: study concept and design, acquisition of data, supervision.

## Conflict of Interest Statement

ET has received research funding from the Swiss National Science Foundation and the Synapsis Foundation, Switzerland. FH received a grant from the Freiwillige Akademische Gesellschaft Basel. R-DS – received a grant form Vifor and serves on an Abvisory Board for Medicine. PF received grants from Parkinson Schweiz, the Jacques and Gloria Gossweiler Foundation, the Freiwillige Akademische Gesellschaft Basel, the Swiss National Science Foundation, the Hedwig Widmer Foundation, and unrestricted grants from Abbvie AG and General Electric. UG received grants from Parkinson Schweiz, the Jacques and Gloria Gossweiler Foundation, the Freiwillige Akademische Gesellschaft Basel, the Gottfried and Julia Bangerter-Rhyner Foundation, the Swiss National Science Foundation, the Camelia Botnar Foundation, and the Hedwig Widmer Foundation, and unrestricted grants from UCB Pharma AG, Abbvie AG, and General Electric.

All the other authors declare that the research was conducted in the absence of any commercial or financial relationships that could be construed as a potential conflict of interest.
